# Mineral nutrient content of infected plants and allied soils provide insight into wheat blast epidemics

**DOI:** 10.1016/j.heliyon.2022.e08966

**Published:** 2022-02-19

**Authors:** Md. Saljar Rahman Chowdhury, Md. Arifur Rahman, Kamrun Nahar, Khondoker M.G. Dastogeer, Islam Hamim, K.M. Mohiuddin

**Affiliations:** a Department of Agricultural Chemistry, Bangladesh Agricultural University, Mymensingh 2202, Bangladesh; b Department of Agricultural Chemistry, Khulna Agricultural University, Khulna, Bangladesh; c Department of Plant Pathology, Bangladesh Agricultural University, Mymensingh 2202, Bangladesh; d Laboratory of Plant Pathology, Tokyo University of Agriculture and Technology, Japan

**Keywords:** Bangladesh, Concentration, Mineral nutrition, Plant nutrients, Wheat blast

## Abstract

Wheat is the second-largest cereal crop in Bangladesh and plays an essential role in ensuring the country's food security. Since 2016, there has been a severe epidemic of wheat blast disease in Bangladesh. This research investigated the nutritional context of wheat blast epidemics by analyzing the infected plants and allied soils. We collected blast-infected wheat plants and allied soil samples from six different severely infected regions of the Meherpur district situated in the western part of Bangladesh. The incidence and severity of wheat blast disease in the sampling fields ranged from 84.78 - 95.11% and 82.06–92.19%, respectively. Among the investigated mineral nutrients in plant samples, the concentrations of sulfur (S), calcium (Ca), magnesium (Mg), iron (Fe) and manganese (Mn) were within the acceptable range of the reference values. In contrast, 50% of the plant samples had insufficient phosphorus (P) concentrations, while others were within the critical range. The potassium (K) and copper (Cu) concentrations in more than 33.5% of plants were within the deficient range. The Si concentrations in half of the tested plant samples were below the acceptable level. However, the boron (B) concentration of around 50% of samples was within the toxic range. The total K, Ca, Zn, Fe, Mn, and Cu concentrations of the soils were lower than the reference values. Based on the interpretation of the available soil test values, the concentrations of S, Fe, Mn, and B in most samples were very low. The concentrations of available P, K, Ca, Mg, Zn and Cu in soil samples were higher than the critical limit. There was a negative relationship between K, S, Ca, Mg, Na and Si concentrations with blast incidence and severity. Therefore, this research suggests that certain plant nutrients such as P, K, Cu, B and Si play a vital role in the wheat blast disease epidemic.

## 1 Introduction

Wheat blast, caused by *Magnaporthe oryzae Triticum* (MoT) fungus, is a very catastrophic disease of wheat. This disease has the potential to cause complete crop failure in the severely affected fields (Kohli et al., 2011; Goulart et al., 1990, 1992). The pathotype Anamorph *Pyricularia oryzae Triticum* was first reported in Parana, Brazil in 1985 (Igarashi 1986). Wheat blast infection was only confined within South America, until February 2016, in which the first outbreak of wheat blast disease occurred in Meherpur, Kushtia, Chuadanga, Jessore, Jhenaidah, Bhola, Barisal, Narail, Magura, and Faridpur in Bangladesh. This was the most devastating shock to wheat cultivation (Malaker et al., 2016; Saharan et al., 2016). Approximately 15% of the total wheat field was infected, and 20% of the overall wheat production was negatively impacted (Malaker et al., 2016). Several comparative genomic studies revealed that the pathogen in the Bangladesh wheat area was clonal and resembled the highly aggressive MoT pathotype of South America (Farman et al., 2017; Malaker et al., 2016). In hot and humid weather, lesions can be noticed not only on the spike but also on all the above-ground plant parts. Due to the lack of complete resistance in cultivars and effective fungicides in the market, the management of this disease becomes difficult (Goulart et al., 2007). In addition, the nutritional status and growth stage of plants during infection can affect the epidemiology of this pathogen (Igarashi 1986; Debona et al., 2014). Because of the complexities of managing wheat blast diseases, different control strategies, increased host resistance and synthetic fungicides need to be examined (Pagani et al., 2014). Yield losses caused by the wheat blast disease can be minimized by growing a few available cultivars with partial resistance combined with fungicide application, integration of nutrient management practices and avoiding the warm and humid environmental conditions favorable to disease development (Rodrigues et al., 2017; Castroagudín et al., 2015).

Plant nutrition is an important part of disease management, yet it is often overlooked. Mineral nutrients affect metabolic processes associated with plant defense and pathogen virulence. Most soils and environments where plants grow are also habitats for plant pathogens. The nutrient status of plants has both positive and negative effects on the incidence of plant disease. Plants deficient in nutrients would be less productive and more vulnerable to several diseases. On the other side, a surplus of nutrients often makes plants more susceptible to disease. Furthermore, various nutrients have specific effects on plant diseases, and certain nutrient elements have a direct and more significant impact on plant diseases than others. There are ample references on the impact of Si, P, Ca, and Mg as an enhancer of resistance to plant disease and their ability to reduce the frequency of wheat blast disease (Cruz et al., 2011, 2015 ).

However, many epidemiological factors of wheat blast; its impact on wheat yield and physiology; the role of plant nutrition in disease outbreaks, and possible treatment options remain unknown. Therefore, further research is needed to understand the influence of mineral nutrients in the wheat blast disease outbreak. Keeping these facts in mind, this study was designed to assess the mineral nutritional perspective of the wheat blast disease outbreak in western Bangladesh. In this study, we determined the concentration of different plant nutrients of wheat blast infecting whole plant and allied soil samples to recognize the role of plant nutrients on wheat blast disease outbreaks in the western part of Bangladesh by comparing them with different reference values.

## 2 Materials and methods

### 2.1 Experimental location

The *Gangni* sub-district of *Meherpur* district is located between 23°44′ and 23°52′ north latitudes and 88°34′ to 88°47′ east longitudes, covering 341.98 square kilometers (Banglapedia, 2021). It is bordered in the north by the Daulatpur sub-district of *Kushtia*, in the south by the *Alamdanga* and *Meherpur Sadar upazilas*, on the east by the *Mirpur* of *Kushtia* district, and on the west by the *Meherpur* Sadar upazila and the Indian state of West Bengal. The primary source of income for the citizens of this region is agriculture (71%) (BBS, 2018). The main agricultural crops in this area are paddy, jute, wheat, tobacco, maize, and vegetables.

### 2.2 The meteorological data to correlate blast disease incidence

In our study, Global Historical Weather and Climate Data collected from Climate Data Online (CDO) of the National Centers for Environmental Information (NCEI0), USA was used to compare the existing climatic conditions of the study area during November 2016 to March 2017 (Weather and Climate, 2021) for blast disease occurrence.

### 2.3 Collection of diseased plant and soil samples

**Figure 1:**
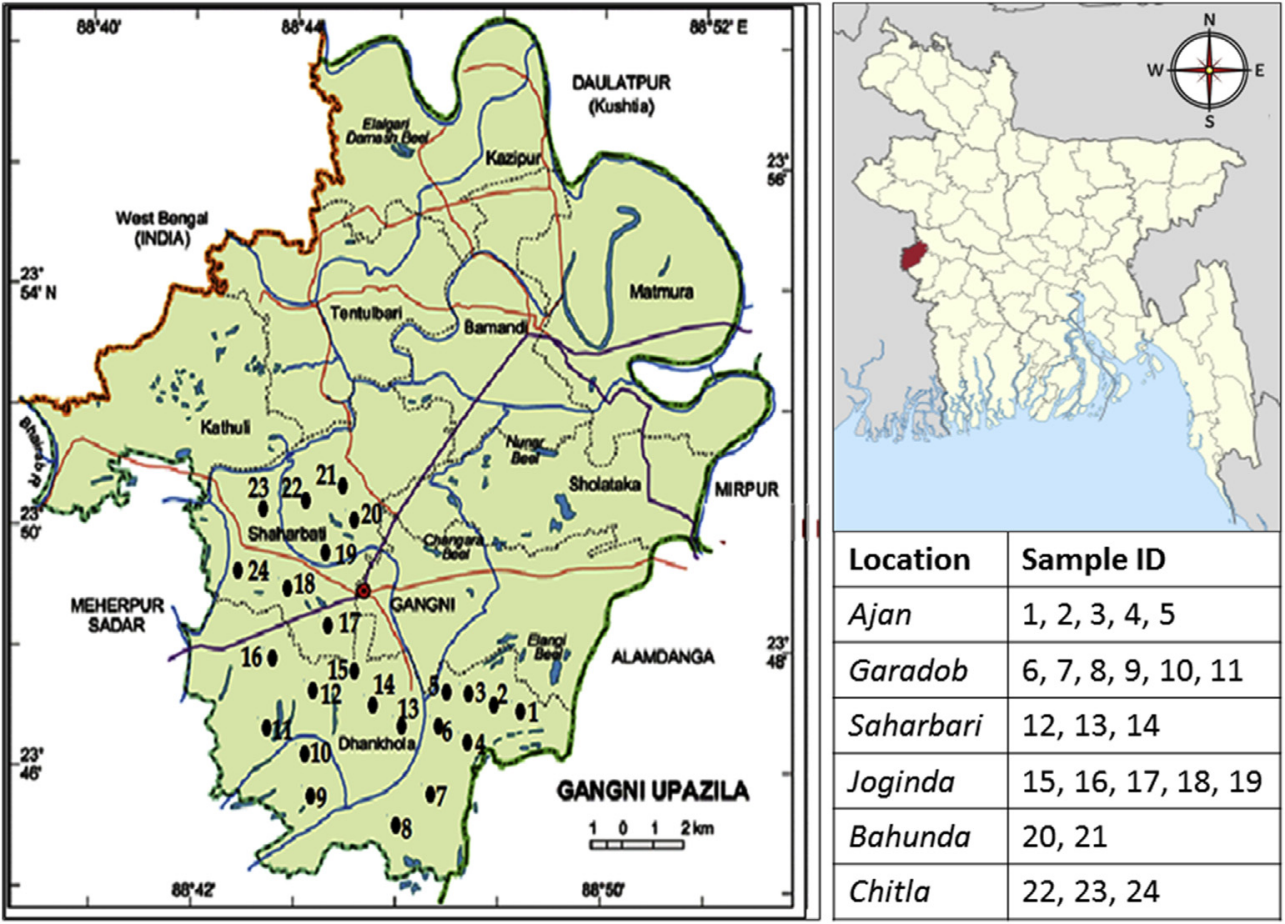
Map showing collection points of wheat plants and allied soil samples.

**Figure 2:**
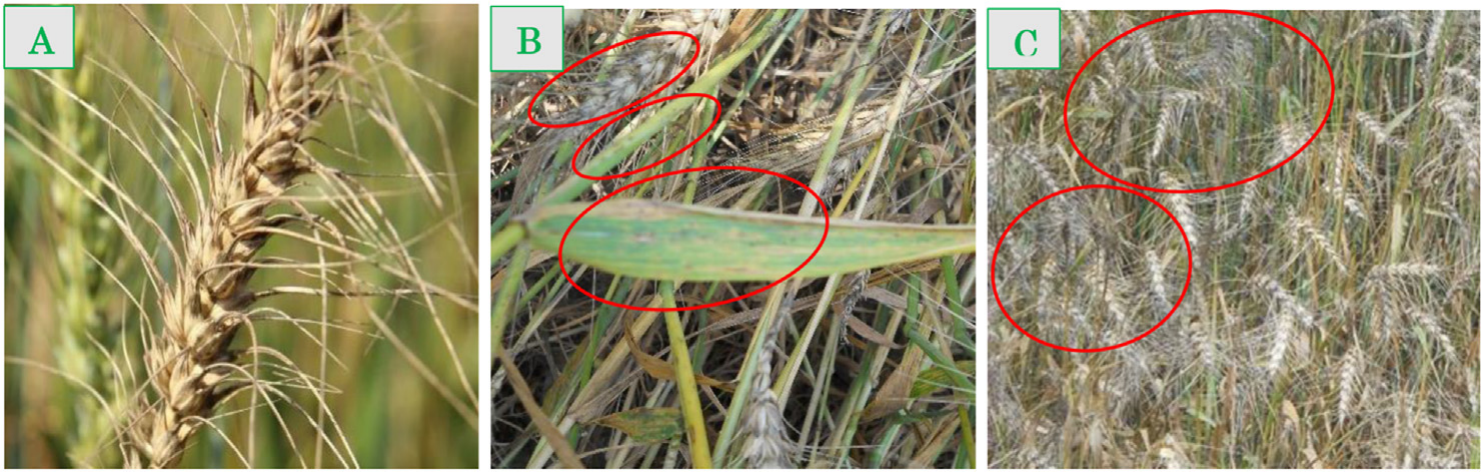
Lesions on spikes, leaves, and stems of wheat blast. A partially infected spike with gray sporulating lesions (A), leaf, stem, and spike with conspicuous yellow chlorotic halos (B), and a large number of blast infected spikes in the field (C).

After complete grain filling, the entire aerial section of the infected wheat plants cv. BARI Gom 24 was randomly sampled with allied soils from 24 severely infected wheat fields (Figure 1) of six individual regions in Gangni upazilla of Meherpur district during February–March 2017. Wheat fields that were cultivated with only *cv.* BARI Gom-24 was selected for sampling, though there was *cv.* BARI Gom-26 in some areas. Farmers in the research areas confirmed that seeds were sown using the broadcasting method and Si was not applied to the designated fields (Surovy et al., 2020). One kilogram of diseased plants showing blast symptoms on the leaves and neck (Figure 2) was collected along with allied soils from each site. In order to estimate yields, a 2 m × 2 m quadrate was set up in the field in 3 replications. Plants within the quadrate were harvested. To determine wheat blast infection in the collected samples, we inspected the samples for bleached (dead) spikes, eye-shaped necrotic lesions with gray centers in the leaves, and symptoms similar to those reported earlier (Malaker et al., 2016; Urashima et al., 2010; Igarashi 1990).

After proper labeling, the samples were brought to the laboratory. An optical microscope was used to examine whether the spike and leaf symptoms of wheat were caused by blast fungus (MoT). Finally, 24 samples from six regions were selected for nutritional studies based on field observations and microscopic testing. Soil samples for soil nutrition analysis were collected from the same locations as the 24 plant samples. All chemical analyses were done at the Department of Agricultural Chemistry of Bangladesh Agricultural University, Mymensingh.

### 2.4 Calculations of disease incidence and severity

Visual estimation on spikes was used to rate disease on a 0–100 scale to calculate disease incidence (Stubbs et al., 1986). The incidence of wheat blast disease in each plant was determined using the modified method of Waller et al. (2002) using the following formula (1)

(1) Disease incidence (%) = Pi×100/Pt

Where, Pi = Number of infected plants, and Pt = Total number of plants assessed. On the other hand, disease severity was calculated following formula (2) (Roy et al., 2018; Tanjina et al., 2019)

(2) Disease Severity (%) = (Spike area diseased / Total spike area) × 100

### 2.5 Preparation of plant extract

Collected and confirmed diseased wheat plant samples were oven-dried (65 °C for 48 h), ground, and then preserved in plastic bottles. The plant extracts were prepared using the wet oxidation technique reported by Singh et al. (1999) and were stored in plastic bottles for further chemical analysis. The plant extracts for silicon analysis were prepared according to the technique Estefan et al. (2013) suggested.

### 2.6 Soil extract preparation

*Available nutrients in soil samples:* The available phosphorus was determined by extracting the sample with a 0.5 M NaHCO_3_ solution (pH of 8.5), as described by Olsen (1954). Exchangeable K, Ca and Mg concentrations in soil samples were extracted using neutral ammonium acetate (1N) as soil extractant. Available S in soil samples was extracted using a 0.01M calcium biphosphate extracting solution. Extraction of available metals (Zn, Cu, Mn, and Fe) was done using DTPA extracting solution as a soil extractant. Available B and Si concentrations in soil samples were extracted using 0.01 M calcium chloride extracting solution following Estefan et al. (2013).

*Total mineral elements:* The soil samples were digested in Teflon containers following the proposed methods of Tessier et al. (1979) to determine the concentration of total minerals. Briefly, 1.0 g of dried soil was heated at 120 °C with a 5:1 mixture of (analytical grade quality) HF and HClO_4_ near dryness. The second addition of HClO_4_ and HF (1:10) were made, and again the mixture was evaporated to near dryness. Finally, 1 mL of HClO_4_ was added to the sample and evaporated till the white fumes appeared. Then the residues were dissolved in 12N HCl and filtrated through Millipore 0.45 μm filter (Whatman no. 42). The filtrate was collected into a plastic container, and the final volume of the solution was made to 50 mL with millipore water.

### 2.7 Determination of the mineral nutrients

Potassium, S, P, Ca and Mg of plant and soil samples were determined following standard methods of analyses (Ghosh et al., 1983; Page et al., 1982; Jackson 1973). Boron was quantified in plant and soil samples using the Azomethine-H technique (Page et al., 1982). The concentrations of heavy metals (Zn, Cu, Fe, and Mn) in plant and soil samples were determined using an atomic absorption spectrophotometer (AAS) (Shimadzo, AA7000, Japan). Silicon was analyzed in plant and soil samples using the spectrophotometric method developed by Estefan et al. (2013).

### 2.8 Statistical analysis

All the statistical analyses were performed using the computer package Mini-Tab version 17 (Minitab Inc, USA). All data fit a normal distribution. The comparison between blast disease-free and blast-infected wheat plant samples for the concentration of various plant nutrients were analyzed in accordance with the guidelines established by Reuter and Robinson (1997).

## 3 Results and discussion

### 3.1 The climatic conditions of blast disease incidence

**Figure 3:**
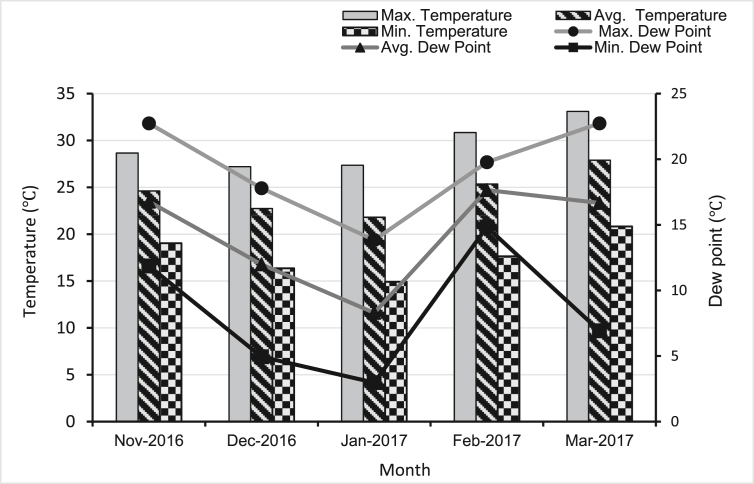
Temperature and dew point of *Meherpur* district during the wheat growing season of 2016–2017.

**Figure 4:**
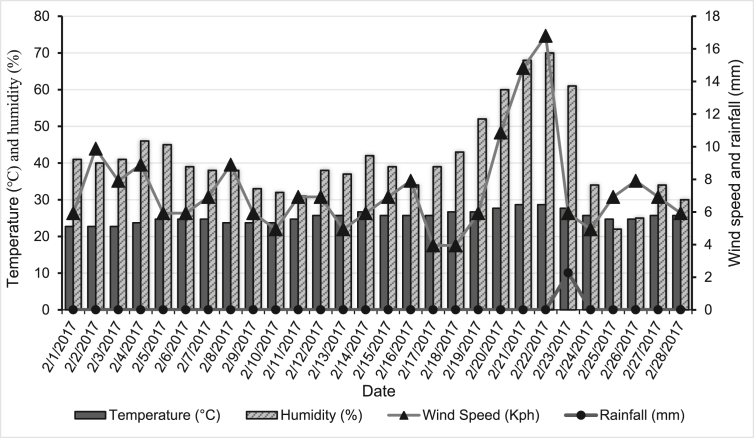
Temperature, humidity, wind speed, and rainfall in February 2017 showing the most probable time for incidence and rapid spread of wheat blast disease.

Wheat blast is a particularly dangerous disease to deal with due to its rapid spread and limited timeframe of opportunity for farmers to implement preventative measures (Chowdhury et al., 2017). In February and March of 2017, a wheat blast pandemic broke out in Bangladesh's *Meherpur* area due to a combination of higher temperature, humidity, dew point, and rainfall as mentioned in Figure 3 and Table 1. Recorded climatic data for the period of mid-February and February 24, when high humidity and wind speed were noted, was suitable for blast disease epidemics in the study location (Figure 4). A previous study showed that seasons of continuous rain with an average temperature of 18–25 °C at the wheat flowering stage followed by a time of sunny, hot, and humid weather are the most favorable conditions for blast infections (Kohli et al., 2011). Temperatures of around 28 °C and high humidity (>90%) have been shown to promote conidia growth in climate-controlled experiments (Alves and Fernandes 2006). At least 10 h of rain and a temperature of 25–30 °C are ideal conditions for infection, according to Cardoso et al. (2008). However, Ha et al. (2012) found that 24 h and a similar temperature range (26–32 °C) is ideal for infection.

**Table 1:** Rainfall, humidity, and wind velocity of *Meherpur* district during the wheat growing season of 2016–2017.

Month	Rainfall (mm)				Humidity (%)			Wind (kmh)		
	Max.	Avg.	Min.	Sum	Max.	Avg.	Min.	Max.	Avg.	Min.
Nov-2016	12.16	0.85	0.00	25.41	95.0	60.8	46	13.8	7.25	2.97
Dec-2016	0.00	0.00	0.00	0.00	62.0	49.2	33	10.87	8.1	3.95
Jan-2017	0.00	0.00	0.00	0.00	53.0	41.1	33	11.86	8.86	4.94
Feb-2017	2.27	0.08	0.00	2.27	70.0	41.1	22	16.8	7.34	3.95
Mar-2017	7.12	0.76	0.00	23.65	77.0	57.0	30	20.76	9.66	3.95

In addition to the above factors, some other factors can be considered in the occurrence of wheat blast disease. In many crops, excessive nitrogen fertilization may lead to an increase in disease severity (Ballini et al., 2013). However, this may not be consistent with results in South America, where early planting is necessary to avoid the rain (Malaker et al., 2016), while late sowing is at higher risk of infection (Mehta et al., 1992).

### 3.2 Incidence and severity of wheat blast disease in the study area

**Figure 5:**
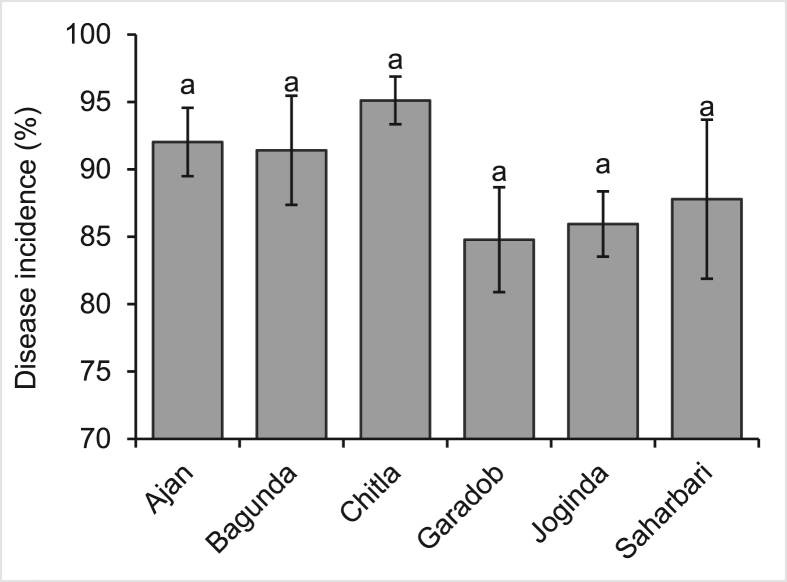
Disease incidence of wheat blast in the respective study area of Meherpur district of Bangladesh. (The values with different letters within a single test crop differ significantly according to Tukey's-test at p < 0.05).

**Figure 6:**
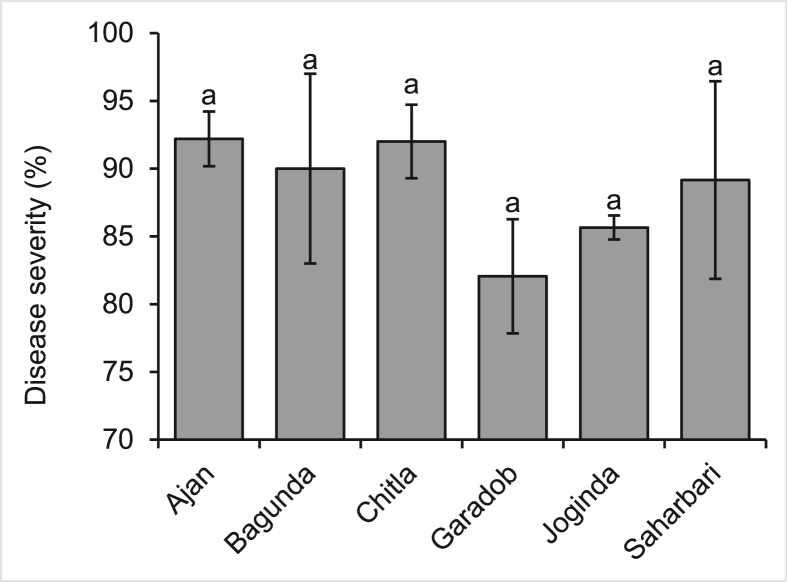
Disease severity of wheat blast in the respective study area of Meherpur district of Bangladesh. (The values with different letters within a single test crop differ significantly according to Tukey's-test at p < 0.05).

Blast fungi are characterized by the formation of asexual spores with a distinct shape composed of three-celled pyriform conidia (Klaubauf et al., 2014). Microscopic investigations indicated gray-colored lesions on both spikes and foliage. This suggests that the fungus found in these lesions is a member of the *Pyriculariaceae*, making it consistent with a prior finding (Islam et al., 2016; Urashima et al., 2010). Wheat blast disease incidence and severity were not significantly different between selected locations of the research area (Figures 5 and 6). Disease incidence varied from 84.78% to 95.11% across the selected locations. *Chitla* had numerically the greatest incidence of wheat plant blast disease, whereas Garadob had numerically the lowest disease incidence (Figure 5). The mean wheat blast disease severity of the study locations ranged from 82.06 to 92.19%. Actually, there was an insignificant variation in the severity of wheat blast disease throughout the research region. However, *Ajan* had numerically the highest severity and *Garadob* had numerically the lowest severity (Figure 6).

Wheat blast disease, caused by *Magnaporthe oryzae* pathotype *Triticum* (MoT), spreads through contaminated seeds, agricultural waste and airborne spores that can travel long distances (CIMMIT, 2021). For these reasons, higher incidence and severity might be found. Another important factor for this high disease incidence is the favorable temperature and rainfall as mentioned in our climatic data. In addition, the same variety of wheat was sampled, which lead to an almost similar and identical amount of disease incidence and severity. As the sampling was done during the last week of February 2017 to early of March 2017, only infected fields were considered, resulting in the selection of severely infected fields. According to a previous study, it was shown that the wheat blast incidence in the *Kushtia* and *Meherpur* districts of Bangladesh ranged from 15 to 43%. In contrast, *Dhanakkhula* of *Gangni*, *Meherpur* had a high blast severity of 20% and a low of 10% (Tanjina et al., 2019).

### 3.3 Macro- and micro-nutrients in wheat blast infected plant and allied soils

The effects of mineral nutrients on plant disease may be attributed to the effects on plant growth, cell walls and tissues, biochemical composition, growth rate of the host, and the pathogen (Colhoun 1973). Crop responses to different mineral nutrients may be favorable or adverse to diseases, depending on disease type, crop species, cultivar, and severity. Balanced nutrition is a factor that increases resistance to plant disease (Fageria 2009).

**Figure 7:**
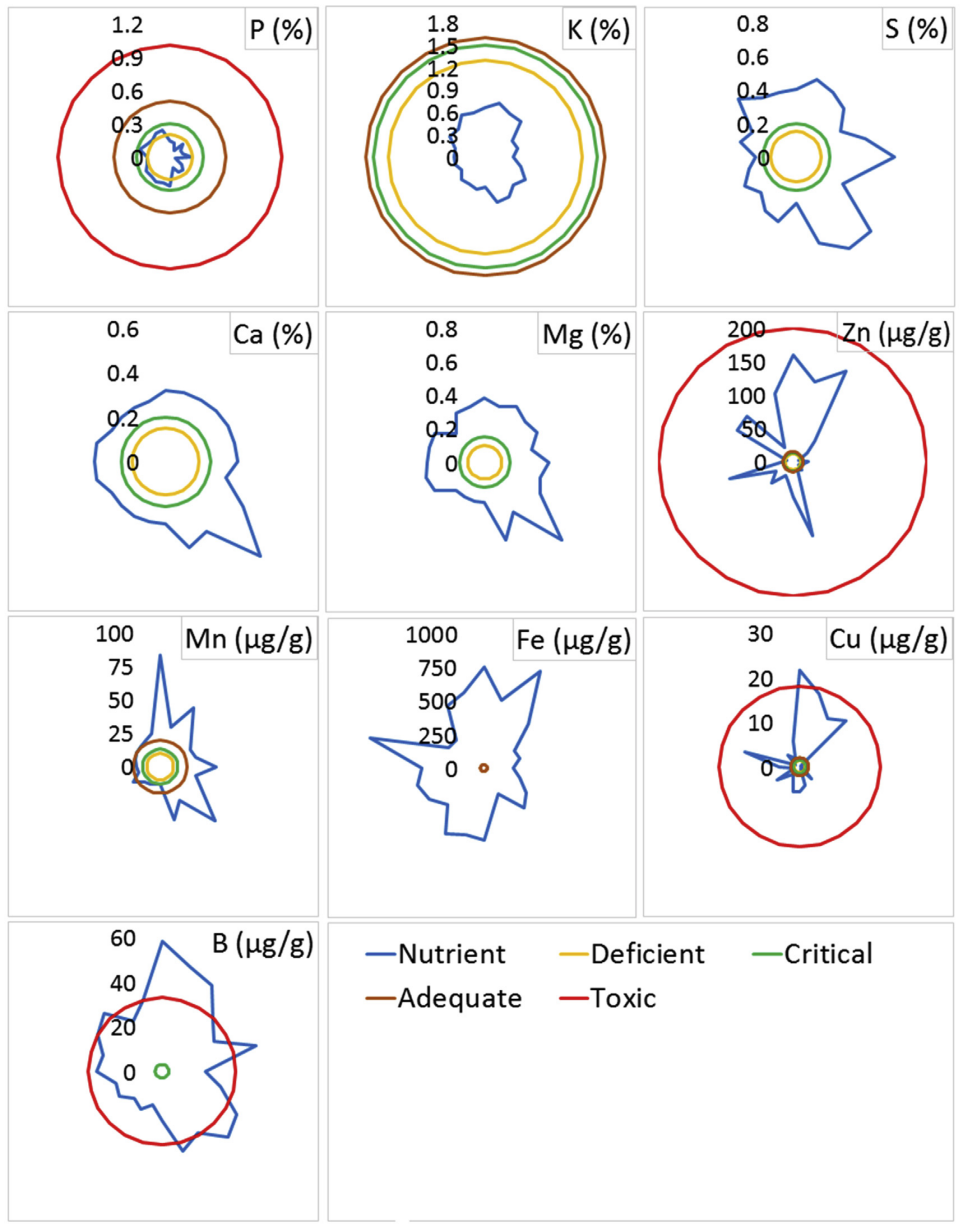
Concentrations of P, K, S, Ca, Mg, Zn, Mn, Fe, Cu, and B in wheat blast infected whole plant samples and their comparison with guideline values.

Phosphorus, a vital plant macronutrient, makes up about 0.2% of a plant's dry weight. It is one of the major limiting factors for plant growth (Schachtman et al., 1998). This study revealed that the concentration of P in the infected plant samples ranged from 0.045 to 0.279%, with an average concentration of 0.182% (Figure 7). According to the reference data proposed by Reuter and Robinson (1997), P concentration in 50% of the infected plant samples was deficient, and the other 50% of plant samples were within the critical range. Among the six regions, the highest P concentration was found in *Bagunda* (0.279%) and the lowest was from *Garadob* (0.045%).

The range of the concentration of available P in the allied soil was 36.2–117.1 μg g^−1^ in different sampling locations, which was very high as per the Fertilizer Recommendation Guide (Guide FR 2012) (Table 2). Similarly, all the soil samples contained a higher amount (average 45% higher) of total P than the average shale value of 0.07% (Table 2). Generally, an adequate supply of P is necessary and beneficial in reducing diseases caused by fungi, where vigorous root development plays a vital role in plant disease prevention (Fageria et al., 2011). Previous studies have shown that P application may boost the magnitude of diseases prompted by Sclerotinia in many garden plants, flag smut in wheat, and bremia in lettuce (Huber, 1980). However, it is generally assumed that P has a direct effect involving organic compounds and metabolic processes for plant health improvement and its role in disease resistance is variable and seemingly inconsistent (Prabhu et al., 2007a; Perrenoud 1990). From many experiments, P enhances disease resistance on many crops against oomycetes, true fungi, and includes resistance against rice blast (Dallagnol et al., 2012; Deliopoulos et al., 2010; Manandhar et al., 1998).

**Table 2:** Concentration of available P, K, Ca, Mg, S, Zn, Mn, Cu, Fe, B, Si and Na in wheat blast infected prior to harvest soil samples.

Sample No.	Region	P (μg g^−1^)	S (μg g^−1^)	K (meq 100g^−1^)	Ca (meq 100g^−1^)	Mg (meq 100g^−1^)	Zn (μg g^−1^)	Fe (μg g^−1^)	Mn (μg g^−1^)	Cu (μg g^−1^)	B (μg g^−1^)	Na (μg g^−1^)	Si (μg g^−1^)
1.	*Ajan*	63.5	0.75	0.21	17.0	1.2	0.79	6.80	0.89	1.32	0.17	51.24	1.25
2.		90.7	0.12	0.27	17.0	1.7	1.54	2.97	0.68	0.74	0.09	48.63	2.06
3.		41.0	1.25	0.22	12.0	1.4	0.29	6.48	0.93	0.85	0.11	57.60	1.30
4.		91.3	0.12	0.20	9.0	1.1	3.36	11.27	3.28	0.61	0.15	33.14	1.95
5.		66.9	0.25	0.25	11.0	1.2	3.12	2.00	1.04	0.46	0.13	33.14	3.03
6.	*Garadob*	36.2	3.25	0.26	9.0	1.0	3.23	2.44	2.90	0.55	0.13	47.81	3.01
7.		68.5	1.50	0.26	11.0	1.2	3.09	2.16	1.15	0.64	0.11	42.92	3.64
8.		73.9	2.50	0.27	10.0	1.2	3.12	2.12	1.26	0.61	0.09	38.19	3.48
9.		69.7	4.50	0.24	18.0	1.9	0.47	2.64	0.78	0.77	0.21	42.92	2.17
10.		65.2	2.00	0.24	17.0	1.7	0.97	2.78	1.25	0.63	0.29	45.37	2.51
11.		76.7	2.50	0.24	17.0	1.6	0.49	2.39	1.50	0.43	0.19	44.55	2.57
12.	*Saharbari*	109.0	4.60	0.26	16.0	2.0	0.54	2.75	1.53	0.66	0.23	36.40	1.99
13.		112.1	2.25	0.24	8.0	1.1	2.91	4.50	0.85	0.75	0.78	43.09	2.01
14.		112.6	0.50	0.30	9.0	1.1	3.16	4.72	0.99	0.89	1.68	48.63	2.07
15.	*Joginda*	106.2	1.00	0.27	8.0	1.1	2.45	5.65	0.75	0.69	0.15	47.98	2.17
16.		112.6	0.50	0.24	8.5	1.1	2.60	4.32	1.14	1.68	0.17	31.87	2.07
17.		82.3	1.00	0.30	17.0	2.0	2.40	6.97	1.16	0.78	0.19	78.80	2.67
18.		94.7	1.50	0.29	17.0	2.0	2.14	2.63	1.50	1.70	0.17	68.20	2.21
19.		66.0	0.25	0.29	18.0	2.1	0.78	4.08	1.48	1.80	0.09	65.75	2.81
20.	*Bagunda*	95.2	4.00	0.33	17.0	2.0	1.29	2.69	1.13	0.45	0.08	62.98	2.37
21.		117.1	1.25	0.25	17.0	2.0	1.64	2.29	3.30	0.63	0.23	70.65	0.88
22.	*Chitla*	115.4	1.00	0.23	17.0	2.0	2.68	1.91	1.41	0.50	0.29	62.49	0.97
23.		92.7	0.75	0.24	18.0	2.0	2.35	2.39	1.46	0.53	0.09	73.91	0.92
24.		115.4	2.32	0.25	18.0	2.0	0.52	8.21	0.69	1.68	0.05	62.49	0.97
**Average**		**86.5**	**1.65**	**0.26**	**14.0**	**1.6**	**1.91**	**4.05**	**1.38**	**0.85**	**0.24**	**51.61**	**2.12**
**Min**		**36.2**	**0.12**	**0.2**	**8.0**	**1.0**	**0.29**	**1.91**	**0.68**	**0.43**	**0.05**	**31.87**	**0.88**
**Max**		**117.1**	**4.6**	**0.33**	**18.0**	**2.1**	**3.36**	**11.27**	**3.30**	**1.80**	**1.68**	**78.80**	**3.64**

**Figure 8:**
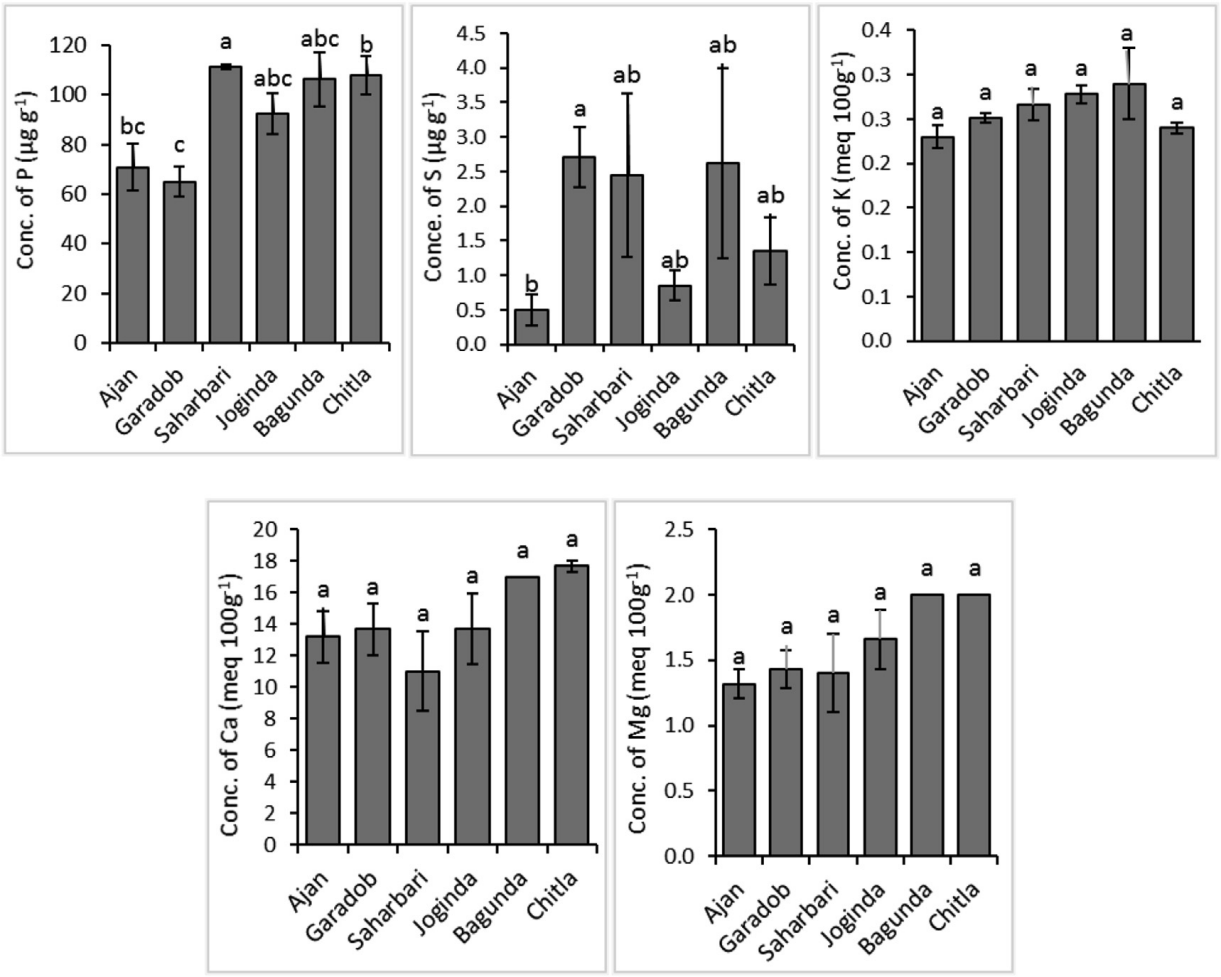
Concentrations of available P, K, S, Ca, and Mg in blast disease infected wheat field soil. (The values with different letters within a single test crop differ significantly according to Tukey's-test at p < 0.05).

Potassium, a plant macro-nutrient, plays an important role in strengthening the cell walls of plants and being involved in tissue sclerenchyma lignification associated with plant resistance to disease (Sugiyanta, 2007). This vital nutrient prevents plant diseases by encouraging the growth of thicker outer walls in epidermal cells (Fageria et al., 2011). While there are a significant number of observations on the function of K and plant diseases, there appears to be little quantifiable evidence on the concentration of K in soil or plant tissue that results in the observed effect on the expression of the disease (Prabhu et al., 2007b). The K concentration of collected plant samples ranged from 0.37 to 0.74%, with an average concentration of 0.50%, which is considered within the deficient range (Figure 7). According to the previous reports, the wheat plant should contain 2.5–5.0% K (Barker and Pilbeam, 2007; Campbell, 2000; Jones et al., 1991). In the respective regions, *Bagunda* site showed numerically higher available K concentration, whereas *Ajan* showed numerically lower concentration (Figure 8). Available K concentration of the sampling location's soil was within the range of 0.20–0.33 meq 100g^−1^ (Table 2). According to Fertilizer Recommendation Guide (Guide FR 2012), concentrations of the available K of 13 soil samples were high, 10 were optimum, and one sample were at very high levels (Figure 8). The total K concentration of the sampling location was within the range of 0.52–1.33%, which was lower than the average shale value of 2.66% (Table 3). K deficient plants appear to be more vulnerable to infection than plants with sufficient supplies of K^+^. While the soil was abundant with K, absorption of K by plants might be inhibited by other factors like susceptible crop variety. As the plant samples were deficient in K, they became susceptible to wheat blast infection due to a reduction in cell turgor which is a physical factor that facilitates the penetration by fungi hyphae (Graham, 1983). K-deficiency also causes the accumulation of soluble amino acids, organic acids, and amines (Fageria et al., 2011). For example, glutamine is exceptionally high in K-deficient plants that stimulate the germination of the fungal pathogen *Pyricularia oryzae* (Graham, 1983).

**Table 3:** Comparison of total mineral nutrients in soil samples with reference values for shale/soil as proposed by Turekian and Wedepohl (1961).

Nutrients	This study (Mean ± SE)	Reference values	% Difference from the reference	Remarks
P (%)	0.10 ± 0.00	0.07	45.1	All the samples were higher than the reference value
K (%)	1.03 ± 0.04	2.66	-61.3	All the samples were lower than the reference value
Ca (%)	1.39 ± 0.09	2.21	-37.0	All the samples were lower than the reference value
Mg (%)	1.95 ± 0.14	1.50	30.1	18 samples higher and six samples were lower than the reference value
Zn (μg g^−1^)	61.21 ± 2.05	95.00	-35.6	All the samples were lower than the reference value
Fe (μg g^−1^)	12484.20 ± 625.17	47200	-73.6	All the samples were much lower than the reference value
Mn (μg g^−1^)	339.70 ± 13.81	850	-60.0	All the samples were lower than the reference value
Cu (μg g^−1^)	16.98 ± 1.34	45.00	-62.3	All the samples were lower than the reference value
B (μg g^−1^)	644.53 ± 22.34	100	544.5	All the samples were higher than the reference value

Plant metabolism is influenced by K, and low K concentrations in the plant may change metabolism, causing favorable conditions for some plant diseases (Mengel and Kirkby, 1978). A sufficient K level plays a vital role in plant disease resistance by increasing phenol concentrations (Prasad et al., 2010). The incidence of various diseases such as sheath blight, sesame leaf spot, rice stem rot, wheat black rust, tea red rust, peanut tikka leaf spot, mungbean and cassava *Cercospora* leaf spot, and *Rhizoctonia solani* seedling rot can be lowered by an appropriate dose of K (Sharma et al., 2005; Huber and Graham, 1999).

Some common Cu deficit symptoms are unhealthy growth, chlorosis/necrosis from the apical meristem to the margin of the leaves, bleaching of younger leaves and ‘summer dieback’ (Rahimi and Bussler, 1973). Copper deficiency varies from plant to plant; wheat, peas, and spinach, which are more vulnerable to Cu deficiency than pea, rye, and rapeseed (Alloway and Tills, 1984). The activity of the enzyme chalcone synthetase, which aids in plant disease resistance by biosynthesis of diverse flavonoids, is generally promoted by Cu compounds (Evans et al., 2007). The Cu concentration of plant samples ranged from 0.23 to 21.65 μg g^−1^ with an average concentration of 5.49 μg g^−1^ (Figure 7). The concentration of Cu in 15 collected plant samples was adequate, eight samples were at deficient levels, and one sample was within the range of toxicity. Wheat blast disease can occur due to Cu deficiency in collected plant samples, which results in increased MoT susceptibility due to poor lignification, delayed leaf senescence, impaired phenol metabolism and soluble carbohydrate accumulation (Marschner, 2012). Previous studies have shown that Cu deficit plants are more vulnerable to fungal diseases than plants with adequate Cu supplies (Evans et al., 2007). The average concentration of available Cu in the allied soil was 0.85 μg g^−1^ (Table 2). According to Fertilizer Recommendation Guide (Guide FR 2012), the available Cu concentrations of nine collected soil samples were at very high level, nine samples were at a high level, four samples at an optimum level, and two samples within the medium level range. The total Cu concentration of the sampling location was within the range of 8.57–30.15 μg g^−1^ (Table 3). However, all the soil samples contained lower than average Cu content on continental earth surface of 45 μg g^−1^ indicating that although available Cu concentration in the soil was higher, total Cu in the infected field soil was not adequate. This condition may be occurred due to the different factors i.e., soil organic matter, oxide type and content, clay type and content, redox potential, and activity of microorganisms that combinedly influence the availability of Cu in soil to the plants (Fageria et al., 2002). The increased level of soil P may be influenced Cu deficiency as this reaction has been linked to dilution effects due to increased growth and the depressive effects of P on Cu uptake (Fageria et al., 2002). Several pathogens have also been documented to be more prevalent in Cu-deficient plants (Fageria et al., 2011).

Boron plays a significant function in phenol metabolism and lignification related to plant defensive pathways (Stangoulis and Graham, 2007). The concentration of B in the plant samples ranged from 15.50 to 58.14 μg g^−1^ with an average concentration of 31.36 μg g^−1^ (Figure 7). However, the B concentrations of 12 infected plant samples were at a toxic level, and 12 samples were within adequate levels. The excess amount of B might encourage blast disease attack because cereals are sensitive to B toxicity and require more minor B for their growth (Goldbach et al., 2002). In contrast, it was reported that the most beneficial effects from B are the reduction of the pathogenic fungus *Plasmodiophora brassicae* Woronin in Brassica species (Stangoulis and Graham, 2007) as the movement of fungal hyphae through the cortex is sometimes prevented by boron (Graham and Webb, 1991). The average concentration of available B in the allied soil was 0.24 μg g^−1^ (Table 2). According to Fertilizer Recommendation Guide (Guide FR 2012), the available B concentration of 8 collected soil samples was at very low level, 12 samples were at low level, two at medium levels, and two samples were within the range of very high level. Total B concentration of the sampling location was within the range of 363.37–794.57 μg g^−1^ (Table 3). However, all the soil samples contained higher B than the average shale value of 100 μg g^−1^.

**Figure 9:**
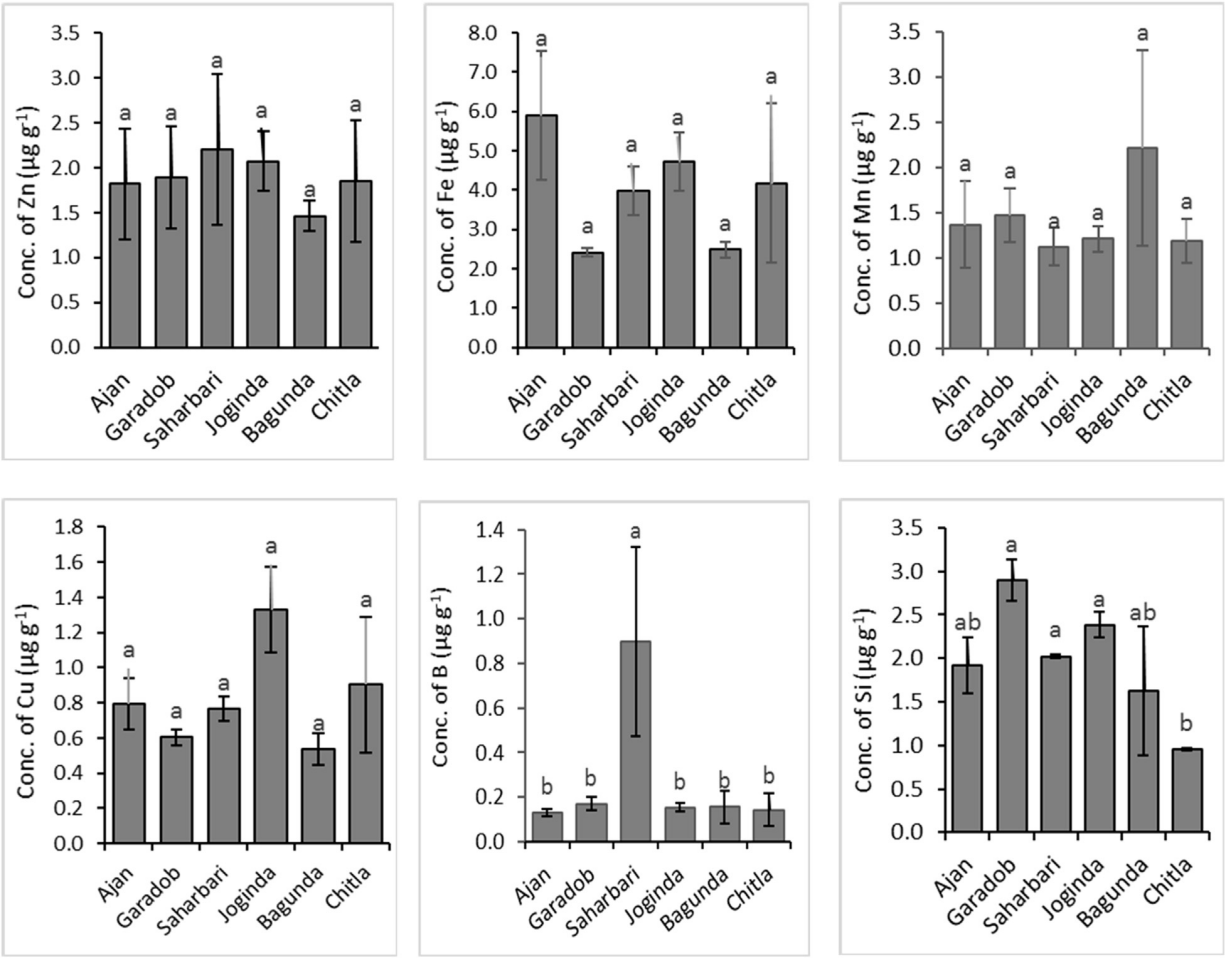
Concentrations of available Zn, Fe, Mn, Cu, Fe and Si in blast infected wheat field soil. (The values with different letters within a single test crop differ significantly according to Tukey's-test at p < 0.05).

Regarding infected soil samples, the value of available Si ranged from 0.88 to 3.64 μg g^−1^ (Table 2). Among the regions, significant variation was found and the highest available Si concentration was found in Garadob (2.89 μg g^−1^) (Figure 9). Currently, there is no standard limit for Si in the soil. Filha et al. (2011) reported that the application of Si fertilizer on wheat plants significantly reduced wheat blast susceptibility by enhancing plant response to infection by *M. grisea.* A lower amount of Si in half of the infected plant sample might be linked to a relatively low amount of available soil Si and might be partially responsible for disease incidence. There are ample references to Si enhancing resistance to different plant diseases. In wheat, Si has been reported to increase resistance to powdery mildew, caused by *Blumeria graminis* f. sp. *tritici* (Bélanger et al., 2003) and spot blotch caused by *Bipolaris sorokiniana* (Domiciano et al., 2010). Nevertheless, Cruz and colleagues found in their investigation that foliar spraying of silicate with artificial inoculation on wheat blast had a minor effect under controlled conditions (Cruz et al., 2011). Silicon deposits or accumulates or acts as an inductor of antifungal compounds under the cuticle of the host plant and thus exerts structural protection (Cai et al., 2009; Datnoff et al., 2007; Rémus-Borel et al., 2005).

Other nutrients (S, Ca, Mg, Zn, Mn, and Fe) also play an important role in plant-pathogen interactions. Nutrients are involved in plant physiology and biochemistry and in many processes that can affect the response of plants to pathogens and reduce the disease severity (Marschner, 2012). The average S, Ca, Mg, Zn, Fe and Mn concentrations of plant samples were 0.405%, 0.32%, 0.34%, 55.63, 456.61 and 29.42 μg g^−1^, respectively (Figure 7). Most of the nutrients in most samples were within the reference adequate range. However, in the case of soil samples, a similar trend was not found. The average concentrations of available S, Ca, Mg, Zn, Fe and Mn in the allied soil were 1.65 μg g^−1^, 14.0 meq 100g^−1^, 1.6 meq 100g^−1^, 1.71 μg g^−1^, 4.43 μg g^−1^ and 1.36 μg g^−1^, respectively (Table 2). According to Fertilizer Recommendation Guide (Guide FR 2012), the available S, Fe and Mn concentration of all the collected soil was categorized as very low, where Ca and Zn concentrations of all the samples were within very high ranges.

The lower amount of available soil-S might be a factor for blast disease incidence in the studied area. Sulfur has a free cysteine pool which assists with disease resistance (Vidhyasekaran, 1988), and non-protein cysteine, the precursor of all S-containing metabolites (Haneklaus et al., 2007). It was found from previous experiments that soil-applied S was shown to increase resistance against different fungal pathogens in a variety of crops under a controlled environment (Klikocka et al., 2005; Wang et al., 2001; Bourbos et al., 2000; Schnug et al., 1995). Sulfur has been found to have a significant impact on the development of fungal diseases, e.g., reduction in leaf spot disease of oilseed rape and stem canker of potato, leading to decreased infection severity (Klikocka et al., 2005). The function of Fe in disease resistance is not well-known in plants (Dordas, 2008), whereas the effect of Mn on plant disease severity has been studied by different researchers (Heckman et al., 2003; Huber and Graham, 1999; Graham and Webb, 1991). The deficiency of Mn decreases the production of phenols and lignins, which provide resistance to fungal pathogens and thus increase disease severity in wheat (Brown et al., 1984).

Thompson and Huber (2007) have stated that the availability of Mn decreases rice blast disease while decreasing the availability of Mn raises the disease correlated with environmental conditions. On the other hand, the concentration of available Mg of 10 samples was within the range of very high, 6 samples were within medium range, 5 in optimum range, and three samples were within the high-level range. However, all the infected soil samples contained Ca, Zn, Fe and Mn in lower than average shale value. Nevertheless, in Mg concentration, only six samples contained a lower amount. The rest of the 18 samples contained a higher amount than the average shale value of 1.5%. Debona et al. (2016) depicted that crops grown with excessive Mg containing soil were more vulnerable to leaf blast as crops experienced the extensive degradation of cellular and photosynthetic components.

### 3.4 Correlation between different nutrients and plant disease incidence and severity

Disease incidence and severity have a close relationship with the availability of nutrients in plants. Among the available nutrients in the wheat, Si showed a very strong negative correlation (r = −0.80) with blast disease incidence and a strong negative correlation (r = −0.66) with disease severity (Table 4).

**Table 4:** Correlation among different plant nutrient content, disease incidence, and severity.

Parameters	Units	Incidence	Severity	P	K	S	Ca	Mg	Zn	Mn	Fe	Cu	Na	B	Si
		%		%					μg g^−1^						%
Incidence	%	1.00													
Severity		0.82	1.00												
P	%	0.18	0.20	1.00											
K		-0.22	-0.19	-0.34	1.00										
S		-0.23	-0.39	-0.32	0.30	1.00									
Ca		-0.51	-0.61	-0.34	0.15	0.52	1.00								
Mg		-0.41	-0.48	-0.41	0.20	0.49	0.88	1.00							
Zn	μg g^−1^	0.35	0.25	-0.19	0.12	0.05	-0.12	0.08	1.00						
Mn		0.03	-0.14	-0.53	0.31	0.47	0.52	0.64	0.43	1.00					
Fe		0.30	0.24	0.25	-0.05	-0.26	-0.12	-0.04	0.38	0.19	1.00				
Cu		0.25	0.21	-0.05	0.32	0.02	-0.04	0.05	0.56	0.50	0.71	1.00			
Na		-0.47	-0.59	-0.56	0.32	0.52	0.62	0.67	0.18	0.46	-0.10	0.03	1.00		
B		0.01	-0.16	-0.55	0.49	0.31	0.38	0.42	0.54	0.74	0.19	0.55	0.59	1.00	
Si	%	-0.80	-0.66	-0.33	0.49	0.29	0.48	0.46	-0.11	0.24	-0.11	0.13	0.59	0.33	1.00

Calcium also showed moderately negative correlations with disease incidence (r = −0.51) and strong correlations with disease severity (r = −0.61) of wheat blast. Moderately negative relationships were found for Mg and Na for both the incidence and severity of wheat blast disease. Potassium and S also showed weak negative correlations (Table 4).

These negative correlations indicate that the decrease of these aforementioned nutrients might increase wheat blast disease incidence or severity. In contrast, it also means that increased disease incidence and severity may decrease the availability of the respective nutrients in wheat plants. In the study, P, Zn, Fe and Cu had a positive correlation with disease incidence and severity, although in some cases, they were not found to be in line with the soil available and total nutrients.

Plants with nutrient deficiency are more susceptible to disease and pests, and supplementing the necessary nutrient(s) may improve their tolerance. The Si concentration of plants is negatively correlated with disease severity and incidence indicating greater resistance to the wheat blast disease which is fully supported by Huber et al. (2012). A high supply of Si significantly lowers the number of lesions on the leaves showing its usefulness as a defense against the disease. Epidermal cells provide a physical barrier to prevent fungal hyphae penetration by depositing Si, phenolics or phytoalexins around the infection peg, which is a common defense mechanism of Si against pathogens (Menzies et al., 1991; Heath and Stumpf, 1986). Decrease in Ca concentrations also increase the disease incidence and severity which may be due to the effects of Ca in the incidence of pathogenic diseases by three mechanisms, i.e., pathogenic invaders at the plasma membrane by changing membrane potential, increases in efflux of low-molecular-weight compounds, and production of Ca-polygalacturonates (Huber et al., 2012). Our data also show that K shortage enhances the vulnerability of host plants to obligatory and facultative parasites. Boron has played a vital role in disease resistance. Boron deficient wheat plants are infected with fungal disease, e.g., powdery mildew as is B adequate ones, and the fungus spreads more quickly across the leaves of deficient plants (Stangoulis and Graham, 2007). Indirectly, micronutrients and beneficial nutrients may influence disease resistance. In our study, Mn and Na showed that their deficiency increases blast disease severity.

## 4 Conclusion

In Bangladesh's *Meherpur* area, wheat production has been destroyed by the catastrophic wheat blast disease, caused by the fungus *Magnaporthe oryzae Triticum*. A significant relationship was found between the blast incidence and nutrient content of the wheat plant in the studied area. Most of the regions severely infected by the wheat blast disease varied from 84.68% to 95.11% and 82.66%–92.19%. High disease intensity may be related to the lower status of K, S, Mg, Na and Si in plant samples. Plant samples examined for nutrients were found to be within the recommended range for wheat development in terms of S, Ca, Mg, Fe, and Mn concentrations. About 50 percent of the infected plant samples were found to be low in P, and a deficient K concentration was found in all of the samples collected. Most of the samples evaluated had Si concentrations that were below the acceptable level. The concentrations of S, Fe, Mn, and B were found to be in the very low range in the majority of the samples analyzed by soil test values. On the other hand, the total K, Ca, Zn, Fe, Mn, and Cu contents of the soil samples measured were lower than the reference value. Despite the greater quantities of P, K, and B found in the soil samples, the plant was unable to assimilate these nutrients. According to the findings of this research, the deficiency of P, K, Cu and Si, and B toxicity may play a role in increasing the disease intensity of wheat blast infection in the studied regions. Although our study provides some insight into the mineral nutritional perspective of wheat blast disease, it is still unknown how MoT infestation is affected by plant available minerals.

## Declarations

### Author contribution statement

Md. Saljar Rahman Chowdhury: Conceived and designed the experiments; Performed the experiments; Wrote the paper.

Md. Arifur Rahman: Performed the experiments; Contributed reagents, materials, analysis tools or data; Wrote the paper.

Kamrun Nahar: Performed the experiments; Analyzed and interpreted the data.

Khondoker M. Golam Dastogeer: Analyzed and interpreted the data; Wrote the paper.

Islam Hamim: Conceived and designed the experiments; Analyzed and interpreted the data; Wrote the paper.

K. M. Mohiuddin: Conceived and designed the experiments; Analyzed and interpreted the data; Contributed reagents, materials, analysis tools or data; Wrote the paper.

### Funding statement

This work was supported by Grants for Advanced Research in Education (GARE), Bangladesh Bureau of Educational Information & Statistics (BANBEIS), Ministry of Education. Government of the People's republic of Bangladesh (Project No.: LS2018659).

### Data availability statement

Data included in article/supplementary material/referenced in article.

### Declaration of interests statement

The authors declare no conflict of interest.

### Additional information

No additional information is available for this paper.
